# Yeast Programed Cell Death and Aging

**DOI:** 10.3389/fonc.2013.00283

**Published:** 2013-11-18

**Authors:** Manuela Côrte-Real, Frank Madeo

**Affiliations:** ^1^Departamento de Biologia, Centro de Biologia Molecular e Ambiental, Universidade do Minho, Braga, Portugal; ^2^Karl-Franzens-Universität Graz, Graz, Austria

**Keywords:** programmed cell death, apoptosis, necrosis, yeast, cell model system

Similarly to metazoans, yeast cells can exhibit several characteristics of apoptosis, including chromatin condensation, DNA breakage, flipping of phosphatidylserine to the outer leaflet of the plasma membrane, accumulation of reactive oxygen species (ROS), and release of pro-death factors such as cytochrome *c* or Endonuclease G from mitochondria. Yeast programed cell death has been shown to occur in response to a variety of stimuli, such as oxidative stress, exposure to acetic acid, and expression of mammalian pro-apoptotic proteins. This program is also inherent to the yeast life cycle, as aged mother cells and cells exposed to pheromone also display an apoptotic and necrotic phenotype. Yeast therefore comprises a conserved core programed cell death process that shares several regulators with mammalian cells, which play major roles in the pathogenesis of human diseases. At the same time, it lacks many of the cell death regulators that have evolved in higher eukaryotes, probably due to the invention of multicellularity. The simplicity of the yeast model allows elucidating the basic molecular pathways of programed cell death without interference from multifaceted regulation, due to various protein isoforms or cellular specificity often observed in studies using mammalian systems. In addition, yeast heterologous expression systems offer the opportunity to exploit the individual functional and mechanistic properties of mammalian apoptotic regulators.

This special issue gathers recent studies enhancing the understanding of PCD and its deregulation, relevant in human pathologies and aging. These include review, mini-review, original research, perspective, and hypothesis and theory articles dealing with the identification of previously uncharacterized proteins and the function of different cellular compartments and organelles involved in PCD and aging, as well as the exploitation of humanized yeasts to untangle the role of apoptotic regulators.

Yeast has long been established as a valuable model system to study conserved biological processes of relevance to human health, and several reviews address the importance of studying endogenous yeast mechanisms to understand human pathologies, particularly cancer and aging. Mollinedo stresses the relevance of lipid rafts in cell physiology and the advantages of the yeast model system to address unsolved questions regarding their role in survival and cell death signaling in mammalian cells, which will impact the design of lipid raft-mediated approaches to treat human pathologies caused by dysfunction of survival and cell death processes (1).

Tosato and co-workers review two yeast models relevant for cancer formation and progression, one mimicking genome instability, a hallmark of cancer, and another metabolic features of cancer cells, including the Warburg effect (2), whereas Mazzoni and colleagues hypothesize that their newly developed yeast clonal life span assay will provide a valuable complement to aging studies (3).

Kitanovic and co-workers showed that intracellular acidification resulting from accumulation of acetic acid in exhausted medium, causes cellular energetic deficiency and nutrient starvation (4). The role of acetic acid, one the main alcoholic fermentation sub-products, as an extrinsic mediator of both processes, and the key function of acetic acid detoxification enzyme Ach1p for mitochondrial functionality, is discussed by Orlandi and co-workers (5).

Oxidative stress is frequently associated with cell death and severe human pathologies. Farrugia and Balzan discuss oxidative stress in yeast, specifically sources of ROS, their molecular targets, and consequences of ROS accumulation, such as up-regulation of antioxidant defenses, activation of both pro-survival and PCD mechanisms, including apoptosis, autophagy, and necrosis, as well as the relevance of ROS in yeast aging (6).

Several articles review the role of organelles in PCD. Guaragnella and colleagues discuss the role of mitochondria and of mitochondrial proteins with an attributed role in the execution and regulation of PCD in yeast, underscoring the use of yeast cells to unravel the mechanisms behind human diseases associated with mitochondrial dysfunctions (7).

An increasing body of evidence shows that organelles other than mitochondria are also involved in PCD and aging scenarios. The function of the endoplasmic reticulum (ER) in PCD is discussed by Austriaco. He identifies the link between the ER and mitochondria during PCD, and the mechanisms leading to ER fragmentation associated with ER stress, as two emerging research areas (8).

It has recently been proposed that peroxisomes can also contribute to oxidative stress, and therefore foster aging and cell death, though through not completely understood mechanisms. Manivannan and co-authors review the current knowledge on the role of peroxisomes in these degenerative processes focusing on data obtained in yeast, and pinpoint future research lines, namely the study of peroxisomal unfolded protein response; the selective inheritance of peroxisomes during replicative aging, and the role of peroxisomal dynamics versus functionality during chronological and replicative aging (9).

Two reviews also address the evolutionary aspects of PCD mechanisms. Shlezinger et al. stress the differences in PCD mechanisms between yeast and metazoans, as well as the similarities and differences of the PCD machinery between single and multi-cellular fungi, highlighting the contribution of filamentous yeast species to apoptosis studies (10). Shresta and Megeney analyze the non-death role of metacaspases in the regulation of cell cycle and proteostasis and protein aggregate formation, and discuss how the cellular utility and roles of the caspase family may have evolved (11). Ren and co-workers studied the relation between checkpoint malfunction and cell death, and suggest that Bir1 plays a concerted role in both the spindle assembly checkpoint and in preventing cell death (12).

Several articles also underscore the use of humanized yeasts to untangle complex biological processes. Van Rossom and co-workers provide an example by using the yeast system to dissect apoptotic properties of the human tumor suppressor protein DFNA5, mapping a domain of DFNA5 that can induce mitochondria-mediated PCD in yeast, as well as a mutation that abrogates it (13). Braun reviews the use of a yeast neurotoxicity model to understand the role of mitochondrial dysfunction in neurodegenerative disorders, particularly their involvement on the prevention or execution of cell death (14). Clapp et al. review the use of genetic screens in yeast using cDNA expression libraries generated from mammalian cells to identify novel PCD regulators, particularly anti-apoptotic components (15).

The data gathered by the studies discussed in this special issue and by many others in the field are promising and foster the use of this simple eukaryotic model system to further unravel the mysteries of cell aging and PCD.
